# Charting the complexity of the activated sludge microbiome through a hybrid sequencing strategy

**DOI:** 10.1186/s40168-021-01155-1

**Published:** 2021-10-15

**Authors:** Lei Liu, Yulin Wang, Yu Yang, Depeng Wang, Suk Hang Cheng, Chunmiao Zheng, Tong Zhang

**Affiliations:** 1 Environmental Microbiome Engineering and Biotechnology Laboratory, The University of Hong Kong, Hong Kong SAR, China; 2 State Environmental Protection Key Laboratory of Integrated Surface Water-Groundwater Pollution Control, School of Environmental Science and Engineering, Southern University of Science and Technology, Shenzhen, China; 3 School of Environmental Science and Engineering, Southern University of Science and Technology, Shenzhen, China; 4 Nextomics Biosciences Institute, Wuhan, China; 5 Department of Chemical Pathology, The Chinese University of Hong Kong, Hong Kong SAR, China

**Keywords:** Long-read metagenomics, Nanopore long reads, Complete genomes, Highly complex metagenomes, Activated sludge microbiome, Haplotype-resolved

## Abstract

**Background:**

Long-read sequencing has shown its tremendous potential to address genome assembly challenges, e.g., achieving the first telomere-to-telomere assembly of a gapless human chromosome. However, many issues remain unresolved when leveraging error-prone long reads to characterize high-complexity metagenomes, for instance, complete/high-quality genome reconstruction from highly complex systems.

**Results:**

Here, we developed an iterative haplotype-resolved hierarchical clustering-based hybrid assembly (HCBHA) approach that capitalizes on a hybrid (error-prone long reads and high-accuracy short reads) sequencing strategy to reconstruct (near-) complete genomes from highly complex metagenomes. Using the HCBHA approach, we first phase short and long reads from the highly complex metagenomic dataset into different candidate bacterial haplotypes, then perform hybrid assembly of each bacterial genome individually. We reconstructed 557 metagenome-assembled genomes (MAGs) with an average N50 of 574 Kb from a deeply sequenced, highly complex activated sludge (AS) metagenome. These high-contiguity MAGs contained 14 closed genomes and 111 high-quality (HQ) MAGs including full-length rRNA operons, which accounted for 61.1% of the microbial community. Leveraging the near-complete genomes, we also profiled the metabolic potential of the AS microbiome and identified 2153 biosynthetic gene clusters (BGCs) encoded within the recovered AS MAGs.

**Conclusion:**

Our results established the feasibility of an iterative haplotype-resolved HCBHA approach to reconstruct (near-) complete genomes from highly complex ecosystems, providing new insights into “complete metagenomics”. The retrieved high-contiguity MAGs illustrated that various biosynthetic gene clusters (BGCs) were harbored in the AS microbiome. The high diversity of BGCs highlights the potential to discover new natural products biosynthesized by the AS microbial community, aside from the traditional function (e.g., organic carbon and nitrogen removal) in wastewater treatment.

Video Abstract

**Supplementary Information:**

The online version contains supplementary material available at 10.1186/s40168-021-01155-1.

## Introduction

Rapid advances in long-read sequencing, also known as third-generation sequencing, achieved by Pacific Bioscience (PacBio) and Oxford Nanopore Technology (ONT), have demonstrated their ability to resolve genome assembly challenges [1–3], e.g., long repeat regions and structural variants [1, 4, 5]. Remarkably, the ONT platform can generate ultra-long reads of up to 2 Mb. On the one hand, the long read lengths could greatly improve the contiguity of genome assemblies [6, 7]. On the other hand, contigs assembled from error-prone long reads have a much higher error rate at the nucleotide level than the short-read-based assemblies. Even with extensive short-read-based polishing, it is still challenging to generate a decent number of high-accuracy sequences, especially for metagenomes with high complexity [8].

Recently, select assemblers have been developed to take advantages of long reads, including the hybrid assemblers Unicycler (for bacterial isolates) [9] and OPERA-MS (for clinical metagenomics) [2], and long-read assemblers Canu [10] and Flye [11]. However, when implementing these assemblers to the complex environmental samples, limitations do apply, e.g., limited feasibility, accuracy, and demanding computational resources. Therefore, an urgent need to develop a new framework that could address the above issues and accurately characterize the high-complexity metagenome remains.

Activated sludge (AS) is one of the primary engineered functional infrastructures associated with public health [12], harboring highly diverse but deficiently characterized microbial communities [13]. The microbial community in the AS is essential for wastewater treatment to remove pollutants (organic carbon, nitrogen, phosphorus, and other toxicants), playing a crucial role in building a sustainable modern society [13]. Therefore, it is important to enhance our understanding to decipher the ecology of the AS microbial community, which may provide new insights into future management strategies of the AS process. Many studies rely on 16S rRNA gene sequencing to capture AS microbial community profiles for linking the microbial composition to potential functions [14–16]. However, connecting the 16S rRNA gene-based microbial profile to the function is very limited due to the challenges of reconstructing HQ metagenome-assembled genomes (MAGs) that included 16S rRNA genes from the AS ecosystem [17, 18].

AS is a diverse environment. Generally, more than 700 genera and a few thousands of operational taxonomic units (OTUs) are observed in the AS [14, 15, 19]. Besides, it is estimated that AS bacterial communities contain about 1 billion bacterial phylotypes, far more complex (at least one order of magnitude greater OTU richness) than those observed in the human gut microbiome [13]. Although sequencing and computational advances, e.g., high-throughput short-read sequencing and accurate assembly/binning tools are available [20], it is difficult to resolve the long repeat regions, as well as to recruit rRNA operons in the genome. These challenges result in the assembly of highly fragmented draft genomes [17, 21], instead of HQ genomes containing full-length rRNA genes. Therefore, the high microbial diversity and complex genome characteristics hinder reference-quality genome reconstruction from AS ecosystems, which limited our capacity to obtain a comprehensive understanding of the AS microbiome.

In this study, we employed a hybrid sequencing strategy and established a feasible iterative haplotype-resolved hierarchical clustering-based hybrid assembly (HCBHA) workflow for high-complexity ecosystems. Benchmarking results based on a mixed mock metagenomic dataset revealed that the average genome quality of the genome reconstructed using the HCBHA workflow achieved Q40, even better than that of recovered from a pure-culture dataset using the short-read-based method. Taking advantages of the high accuracy of Illumina short reads and the large span of ONT long reads, we reconstructed 557 bacterial and archaeal genomes, including 14 complete and 111 high-quality genomes containing full-length rRNA operons from a deeply sequenced, highly complex AS sample. The contiguity (in terms of N50) of these retrieved 557 genomes was improved by an order of magnitude when compared to a short-read-based method. Leveraging the high-contiguity MAGs (average N50 = 574 Kb), we reconstructed the keystone metabolic pathways and identified various biosynthetic gene clusters (BGCs) harbored in the AS microbiome from a wastewater treatment plant (WWTP). The wide range of BGCs highlights the potential versatility of the AS microbiome.

## Methods

### Activated sludge sampling and DNA extraction

AS samples were collected on October 5, 2018 (AS1), and February 1, 2019 (AS2), from the aeration tank at Shatin WWTP (114.21 E, 22.41 N) and transported to the laboratory for further treatment within two hours. For AS1, the DNeasy PowerSoil Kit (Qiagen, Hilden, Germany) was used to extract total DNA from 2-mL AS samples. For AS2, DNeasy PowerSoil Kit and FastDNA Spin Kit for Soil (MP Biomedicals, Santa Ana, CA) were individually used to extract genomic DNA from the same volume (2 mL) of AS sample following the manufacturer's protocols. The sequenced Illumina data generated from the above kits were named “Qiagen dataset” and “MP dataset”. A NanoDrop 1000 spectrophotometer (Thermo Scientific, Wilmington, DE, USA) and Qubit 2.0 Fluorometer (Life Technologies, Carlsbad, CA, USA) were used to determine the DNA purity and concentrations, respectively.

### Constructed mock datasets

To establish the feasibility of the HCBHA workflow and assess the accuracy of generated MAGs, two mock datasets were constructed. The original mock community data (ZymoBIOMICS Microbial Community Standards, Zymo Research Corporation, Irvine, CA, USA) was downloaded from Nicholls et al., including Illumina data of 8 bacterial isolates and Zymo CS Even long-read dataset generated on a GridION platform [22].

In this study, we randomly subsampled 4-Gb-long reads and approximately 100X short reads for each bacterial isolate (in total, 3 Gb) to construct the first mock dataset (hereafter referred to as the “Mock dataset”). The second simulated dataset included short reads of Zymo CS bacterial isolates and Shatin AS sample (12 Gb, each) and long reads of Zymo CS bacterial isolates and Shatin AS sample (14 Gb, each; hereafter referred to as the “Mock-STAS dataset”).

### Illumina and Nanopore sequencing and primary processing

In our study, only DNA extracted using the DNeasy PowerSoil Kit was suitable for the construction of a long-read library due to the quality requirements for Nanopore sequencing. For AS1, the total genomic DNA was sent to the Chinese University of Hong Kong (Hong Kong, China) to generate long reads on PromethION using one flowcell. For AS2, the total DNA extracted with the DNeasy PowerSoil Kit was divided into two parts. One part of the above-described DNA and DNA extracted with FastDNA Spin for Soil Kit were sent to Novogene Company Limited (Beijing, China) for shotgun sequencing to generate high-accuracy reads in 2 × 150 mode on Illumina HiSeq sequencer (Illumina, CA, USA). The other part of the DNA (extracted with a DNeasy PowerSoil Kit) was sent to Nextomics Biosciences Institute (Wuhan, China) to generate long reads on the PromethION platform using 3 flowcells.

Illumina short reads with an average base-quality ≥ 30 and Nanopore long reads with length ≥ 1 Kb were retained for downstream analysis. In total, we obtained 514 Gb of short reads (263 Gb for MP dataset and 251 Gb for Qiagen dataset) and 267 Gb of long reads (1 flowcell of AS1 and 3 flowcells of AS2) with an average read accuracy of 86.7% (Q8.76) (Additional file 1, S1).

### Hybrid assembly workflow for highly complex ecosystems

**Fig. 1:**
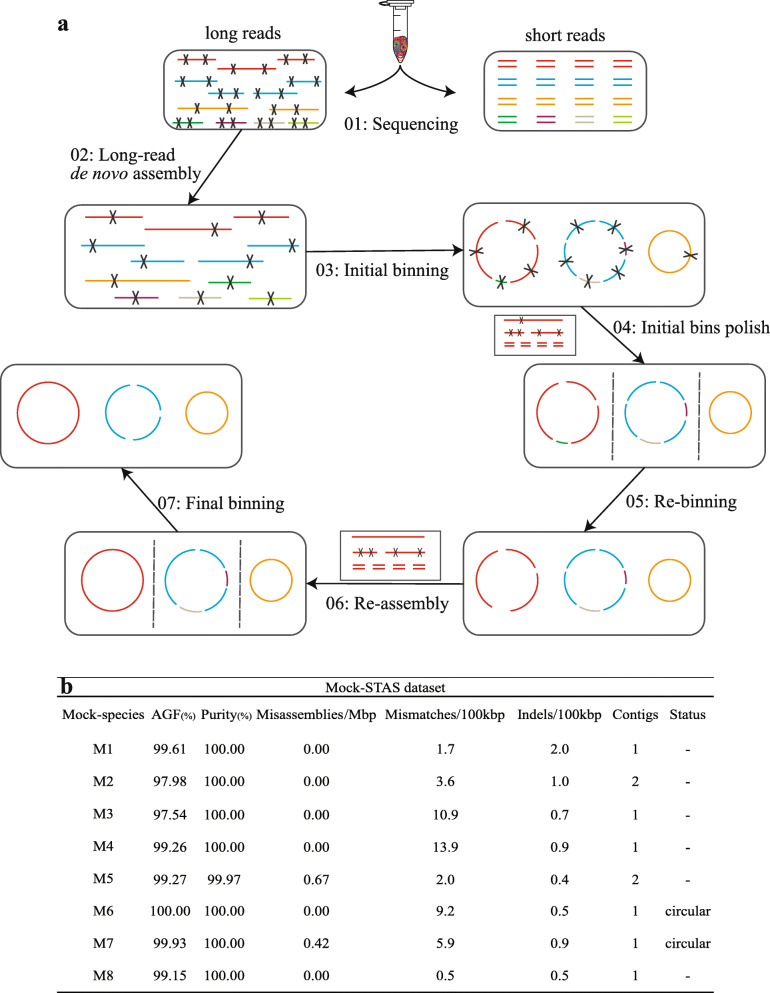
Bioinformatics workflow of the HCBHA approach for complex ecosystems and the evaluation using a mock community. **a** Bioinformatics framework of genome reconstruction from highly complex metagenomes. Dash lines indicate the genome was hybrid-assembled individually. **b** Genome reconstruction performance using the Mock-STAS dataset that combined the 8-species mock sequencing dataset and a subset of activated sludge sequencing dataset

An overview of the HCBHA bioinformatics workflow for a high-complexity ecosystem can be found in Fig. 1a. Seven steps and various tools were integrated into the framework to reconstruct (near-) complete genomes from the AS sample. Briefly, the HCBHA workflow first de novo assembles the low-accuracy long reads (obtained in step 1) using Flye (v2.4.2) [11] with the meta mode [23] (step 2), then clusters the assembled low-accuracy contigs (step 3), followed by improving contigs accuracy (step 4), amending bin clusters (step 5), enhancing assembly contiguity (step 6), and finally promoting bin accuracy (step 7). A detailed description of the workflow is provided in the supplementary files (Additional file 2, Method part).

### Evaluation of the HCBHA workflow

The long and short reads of the two constructed Mock and Mock-STAS datasets were analyzed by the HCBHA workflow. Detailed evaluation of the workflow followed our previous work [24]. Aligned genome fraction (AGF), purity (1-unaligned length/reference genome length) [2], misassembly rate, and indels rate of the reconstructed genome were calculated using the matrices obtained from QUAST (v5.0.2) [25] results.

Meanwhile, SPAdes (v3.13.0) [26] was used to assemble the eight bacterial isolate genomes using default parameters, and metaSPAdes [27] was used to assemble the mixed 8 bacterial metagenomic data, followed by the binning process using “binning (--metabat2 [28] and --maxbin2 [29])” module of MetaWRAP (v1.1) [30]. The quality scores of the assembled genomes were estimated using the script *fastmer.py* (https://github.com/jts/assembly_accuracy).

### Iterative HCBHA approach for the deeply sequenced, highly complex activated sludge sample

Initially, the HCBHA workflow was employed to use all the sequenced long- and short-read datasets for genome reconstruction; however, the demand for computational resources was extremely high. To ensure the feasible processing of such big sequencing data at the commonly available computational capacity (e.g., 256 Gb RAM and 48 threads), we randomly subsampled a total of 90 Gb sequence data as a new attempt to re-start the hybrid assembly process. Among the 90 Gb dataset, 30-Gb-long reads were from the total 267-Gb-long read dataset, 30-Gb-short reads were from the 263-Gb MP dataset, and 30-Gb-short reads were from the 251-Gb Qiagen dataset.

To retrieve more MAGs and improve reads utilization, we employed an iterative strategy to exploit the HCBHA workflow further. Briefly, the reads assigned to qualified MAGs were first excluded, then the HCBHA workflow was employed to reconstruct MAGs from the remaining dataset repeatedly following the methodology described in our previous study [24]. For the highly complex AS sample, qualified genomes were defined as MAGs with completeness of more than 90%, contamination of less than 10%, and contig number was less than 30. The completeness and contamination of reconstructed MAGs were assessed using CheckM (v1.0.80) with the lineage-specific workflow [31]. In each genome reconstruction iteration, MAGs with completeness of more than 50% and contamination of less than 10% were retained. After 11 iterations, the hybrid assembly process stopped as the relative abundance of reconstructed qualified MAGs was less than 2%.

We also performed the last HCBHA iteration to remove reads assigned to all collected qualified MAGs using our 9-year time-series short-read data [32]. Finally, quality-filtered MAGs from each hybrid assembly iteration were collected together and dereplicated by dRep [33] using stringent parameters “-pa 0.90 -sa 0.97 -nc 0.65”.

### Taxonomic assignment and genome annotation

MAGs were classified using GTDB-Tk (v1.1.0, gtdbtk classify_wf) based on the database GTDB RS89 [34, 35]. Genome features, including 5S, 16S, 23S rRNA operons, and tRNA were identified using Prokka (v1.14.5, --kingdom Bacteria or --kingdom Archaea) [36] with dependencies BioPerl, GNU Parallel (20161222) [37], Blast+ (v2.9.0) [38], Prodigal (V2.6.3) [39], aragorn (v1.2.38, for tRNA features), barrnap (v0.9, for predicting rRNA), and HMMER3 (v3.3).

EnrichM (v0.5.0, https://github.com/geronimp/enrichM) was used to explore the metabolic potential for the 39 HQ/complete MAGs with relative abundances of more than 0.2% in the AS system. For HQ/complete MAGs, only pathways with 100% genes identified in the module were considered complete.

Metabolic functional traits of all reconstructed MAGs were profiled by METABOLIC (v3.0) [40] with METABOLIC-C.pl. The parameters “-m-cutoff 1.00” and “-m-cutoff 0.75” were selected for 125 HQ/complete and 432 medium-quality (MQ, completeness ≥ 50% and contamination ≤ 10%) MAGs, respectively. BGCs were identified using antiSMASH (v5.0) [41] with full-featured analysis. “Others” indicated the collection of some rare BGC types identified in the present AS ecosystem, and all the BGC types followed the antiSMASH nomenclature. All the predicted open reading frames (ORFs) were aligned using blastp against the structured antibiotic resistance gene (SARG) database [42] to identify the ARG-carrying genomes with the cutoff of at least 70% amino acids similarity and 70% query coverage.

### Illumina reads assembly and binning

Clean short reads from the MP and Qiagen datasets were combined and assembled using megahit (v1.2.1-beta) [43] with the option “--k-min 27 --k-max 127 --k-step 10”, and then binned using MetaWRAP [30] with the same parameters as mentioned above.

## Results

### Recovering high-accuracy and single-contig genomes from mixed mock community datasets

Two constructed datasets (Mock and Mock-STAS) were used to explore the feasibility and performance of the HCBHA workflow (Fig. 1a). The mock datasets included 8 bacteria with known reference genomes and were considered as a comparative standard for evaluating the accuracy of retrieved MAGs. The main idea of the workflow is to split the long-read and short-read metagenomic datasets into many single microbial datasets. Thus, the complicated metagenomic assembly process could be sub-divided into many efficient assembly tasks for each microbial candidate bin.

For the Mock and the Mock-STAS datasets, the HCBHA workflow demonstrated improved genome reconstruction performance. In detail, seven out of eight bacterial species in the Mock dataset were assembled into single-contig genomes, with three representing full circular genomes. Another assembled genome contained only two contigs each. The average AGF (aligned genome fraction) and purity [2] of the eight genomes were 99.04% and 99.94%, respectively (Additional file 1, S2). Seven assembled genomes displayed no mis-assemblies, and the remaining one displayed a misassembly rate of 0.84/Mb. Notably, similar genome reconstruction performance was also observed using the complex Mock-STAS dataset (Fig. 1b). Among the 8 known species in the mock community, six genomes had single-contigs, the other two had two contigs each, and the average AGF and purity were 99.09% and 100.00%, respectively (Additional file 1, S2).

Besides the appraisal of the hybrid-assembled MAGs with the reference genomes in the mock community, we also compared the hybrid-assembled genomes with short-read-only assemblies. Short reads from the Mock dataset were assembled by metaSPAdes [27], followed by genome binning using metaWRAP. The average AGF and purity of the 8 short-read-assembled genomes were only 82.59% and 85.34%, respectively, with an average contig count of 56 (Additional file 1, S2). Although eight datasets from bacterial pure cultures were assembled individually using SPAdes [26], the results were inferior to hybrid assemblies (Additional file 1, S2). Remarkably, both the hybrid-assembled genomes recovered from the Mock and Mock-STAS datasets demonstrated high sequence accuracy, with a median genome quality score of Q40.5 and Q39.2, respectively (Additional file 1, S2). The best genome quality of reconstructed genome using our approach was Q45.4, even better than short-read-only assemblies (Additional file 1, S2 and Additional file 2, Fig. S1a and b). Overall, genomes reconstructed from metagenomes using the HCBHA workflow manifested benefits in every assessment category (contiguity, sequence quality, completeness, etc.) than even pure culture-based short-read-only assemblies. Benchmarking results revealed that the developed workflow is a powerful approach when reconstructing high-accuracy and high-contiguity genomes from highly complex metagenomic datasets.

### Iterative haplotype-resolved HCBHA approach for the deeply sequenced, highly complex AS sample

Compared to conducting the HCBHA workflow using the subsampled datasets, the assembly process using the full datasets required much more computational resources. For instance, it took 2.4-Tb RAM and 24,000 CPU hours when running long-read de novo assembly (step 2, 267-Gb-long reads) of the workflow. Meanwhile, assembling the full dataset could increase assembly interference due to the high microbial diversity in the complex ecosystem. Therefore, subsampling and iterative strategies [24] were recommended for the HQ MAGs reconstruction process from deeply sequenced, highly complex microbiomes, such as activated sludge samples. Leveraging the above versatile iterative HCBHA approach, we reconstructed 552 bacterial and 5 archaeal MAGs using 267-Gb Nanopore long-read and 514-Gb Illumina short-read data of the deeply sequenced Shatin AS microbiome.

**Fig. 2:**
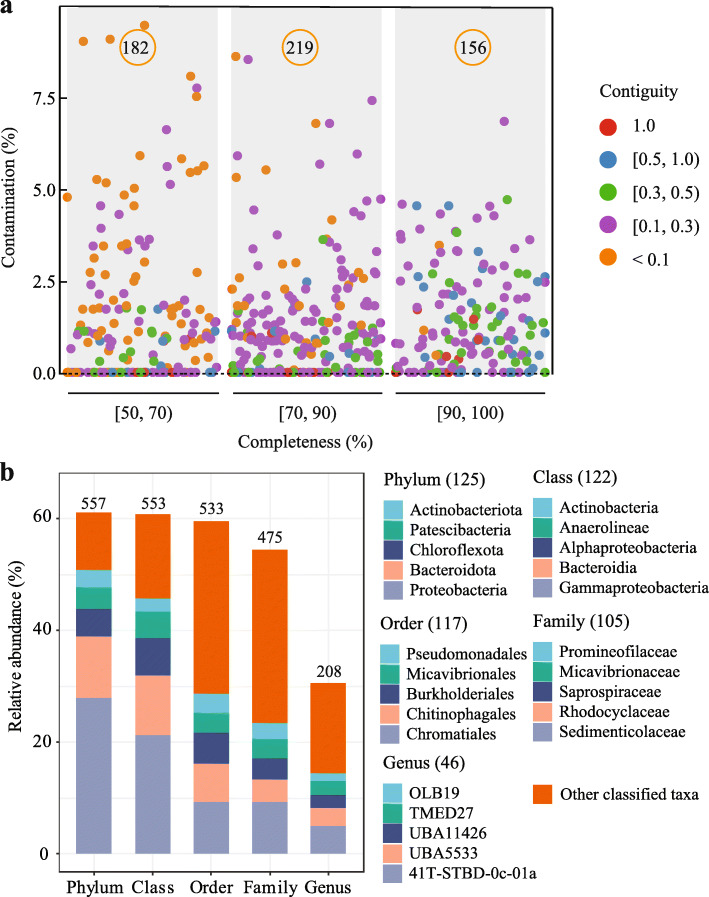
Reconstructed genome quality and taxonomic assignments of the most prevalent activated sludge MAGs. **a** Genome quality overview of the reconstructed 557 MAGs from an activated sludge sample. Numbers in the orange circles indicate the MAG number in three completeness ranges. **b** Community composition of the activated sludge microbiome. Only the top 5 common lineages were shown, with the remaining classified taxa grouped as “Other classified taxa”. Values labeled on top of the bars represent the total number of classified MAGs. Numbers in the brackets of the legend indicate the number of classified HQ/complete genomes for each taxonomic lineage

Integrating with 267-Gb-long reads and leveraging the iterative haplotype-resolved framework, we improved the genome representation in the sample. The reconstructed 557 MAGs represented 61.1% of the microbial community, whereas only 483 MAGs representing 48.0% of the communities were reconstructed using short-read-based method (Additional file 2, Fig. S2a). The retrieved 557 MAGs included 14 closed/complete, 111 HQ, and 432 MQ genomes (Additional file 1, S3). The average contig number was 34, with an average N50 of 574 Kb (~ 10X and ~ 16X improvement compared with short-read-assembled MAGs in this study and by Ye et al. [18], respectively) (Additional file 2, Fig. S2a and b), suggesting that we assembled highly contiguous genomes from this complex ecosystem. Here, we defined contiguity as the percentage of the longest contig in the assembled genome. The reconstructed high-completeness (≥ 90%) genomes were also high-contiguity (Fig. 2a). These HQ and high-contiguity MAGs shall provide near-full characterization of the encoded functional potential of AS in the WWTP.

### Diversity of the MAGs from the activated sludge microbiome

The taxonomic analysis of the 557 highly contiguous MAGs demonstrated that Shatin AS harbored a diverse group of microorganisms, spanning 1 archaeal and 36 bacterial phyla. Most of the MAGs were assigned into the phylum *Proteobacteria* (181 MAGs), *Bacteroidota* (82), *Myxococcota* (51), *Patescibacteria* (47), *Chloroflexota* (46), *Planctomycetota* (37), *Actinobacteriota* (22), *Verrucomicrobiota* (13), and other phyla (Additional file 2, Fig. S3). Not surprisingly, the number of classified MAGs decreased from the phylum (557) to genus (208) and species level (13) (Fig. 2b, Additional file 1, S3). However, the extremely low classification percentage at the species level revealed that the majority (97.7%) of these MAGs did not have a reference genome in the GTDB database (R89), highlighting the novelty of the genomes obtained in this study.

Of these 557 MAGs, 14 MAGs were identified as complete genomes, including 10 circular MAGs with reduced-size genomes, although their completeness was below 90%. The above smaller genomes were affiliated to three bacterial phyla *Patescibacteria* (6), *Myxococcota* (1) and *Proteobacteria* (1), and an archaeal phyla *Nanoarchaeota* (2). The identified 111 HQ genomes met the stringent criteria of MIMAG [44], encoding full-length rRNA operon with high completeness (≥ 90%) and low contamination (≤ 5%). The average N50, completeness, and contamination of the 125 (111+14) HQ/complete MAGs were 1.186 Mb, 93.4%, and 1.3%, respectively (Additional file 2, Fig. S4). These 125 reference-quality genomes accounted for up to 27.1% of all AS microbiomes based on the read mapping method, which was consistent with the single-copy marker gene-based method (28.7%). Notably, full-length 16S rRNA genes were identified in 410 MAGs (74% of total MAGs), suggesting that the long reads, to a great extent, could resolve the limited recovery of 16S rRNA genes in the short-read-based assembly approaches.

### Genome-scale metabolic reconstruction

**Fig. 3:**
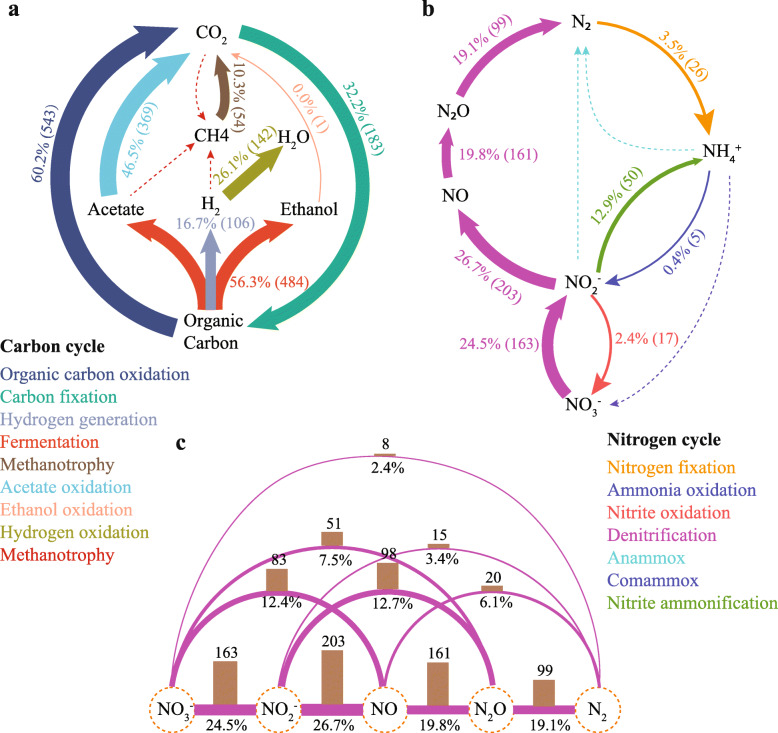
Core metabolic potential (carbon and nitrogen) associated with the functionality of wastewater treatment plants in the activated sludge microbiome. **a**, **b** Carbon and nitrogen cycles in activated sludge. Pathways with no genomes identified are marked by dashed lines. **c** MAGs involved in the high-modularity denitrification process. The numbers and percentages represent the counts of recovered MAGs presumably capable of such reaction and their total relative abundance in the activated sludge microbial community, respectively. All the directions of the denitrification transformation go from left to right

**Fig. 4:**
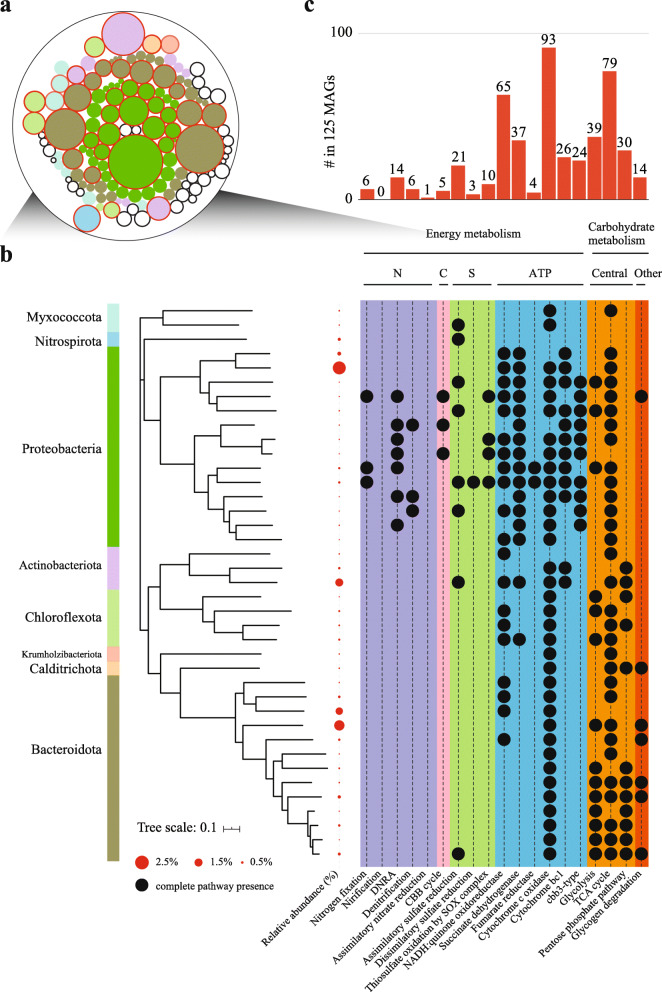
Metabolic potential profiles of the reconstructed HQ AS microbial communities. **a** Taxonomic distribution of reconstructed 125 HQ/complete MAGs. Genome circles with a colorful background stand for the phyla harboring high abundant genomes (relative abundance > 0.2%, represented by the red circles). The size of the genome circles stands for the relative abundance of MAGs. Circles outlined in red are those 39 MAGs used for plotting the phylogenetic tree below. **b** Functional profile of the 39 most abundant HQ/complete MAGs. The presence of complete pathways is shown in solid black dots. **c** Bar chart indicates the total number of MAGs harboring the complete pathway among the 125 HQ/complete MAGs

Removing pollutants from wastewater and protecting the water quality of the receiving water body are paramount for the engineered AS ecosystems. Much of it is accomplished by the versatile microbes [45]. As expected, many MAGs were identified to harbor the genes associated with the carbon and nitrogen cycles (Fig. 3a and b). The main KEGG modules of the most abundant microorganisms (relative abundance > 0.2%, Fig. 4a, b) showed that, among the HQ/complete MAGs set, 79 HQ MAGs were found to encode full genes for the TCA cycle (Fig. 4c). A majority (97.5%) of the recovered MAGs in the present study showed the capability of organic carbon oxidization and fermentation (86.9% of the recovered MAGs), as well the oxidation of low-molecular-weight organic acids (e.g., acetate) (66.4% of the recovered MAGs) (Fig. 3a). Additionally, 54 MAGs, accounting for 10.3% community abundance, exhibited methanotrophic potential (Fig. 3a). The high-abundance of methanotrophs in the system suggested a great possibility to attenuate methane emission from the WWTP.

A function of a WWTP is the removal of nitrogen through biological nitrification and denitrification [46]. Nitrite was the most frequently identified electron acceptor candidate within the nitrogen cycle as 36.4% of MAGs (203) encoded enzymes that had the potential of catalyzing nitrite to nitric oxide. Five MAGs were classified into the genus *Nitrosomonas*, an ammonia oxidizer that can oxidize ammonia to nitrite (Fig. 3b). None of these 5 MAGs could be assigned to the species level and thus represent potentially novel species. This could be an indicator that our current understanding of the AS ecosystem including microbes and their functional groups is still limited.

In addition, hydrogenases were identified in 106 MAGs (16.7% community), and 142 MAGs presented the capacity for hydrogen oxidation. Among them, 76 MAGs encoded both genes for hydrogenases and hydrogen oxidation (*hox*) (Additional file 1, S4). However, the expression level of the associated functional genes and estimation of the amount of hydrogen released from the AS process require further investigations. Besides, a diverse group of microorganisms are potentially involved in the different steps of the denitrification process (Fig 3b, c). However, only eight annotated genomes encode the full set of enzymes capable of reducing nitrate to nitrogen gas (Fig. 3c), and the MAGs harboring one or a few steps in the denitrification process were more prevalent in the Shatin WWTP (Fig. 3b, c). Similar pattern was also observed for sulfur cycle reactions (Additional file 2, Fig. S5), and other processes, e.g., the process of organic carbon oxidation (Additional file 1, S4) and nitrification (no comammox process) (Fig. 3c).

### Lineage-resolved secondary metabolites biosynthesis encoded within the AS microbiome

Microorganisms can produce a diverse of secondary metabolites (SMs, natural products), which have been widely utilized as antibiotics, insecticides, antitumor agents, and other beneficial natural products [47, 48]. SMs are not necessarily directly involved in survival but represent essential functions to mediate interactions with other microorganisms and the environment [49, 50]. The production of SMs might play a critical role in the densely populated AS ecosystem, allowing them to better interact with the neighboring niches. BGCs normally carry a few physically collocated genes that encode the production of SMs; thus, it is difficult to identify the full gene cluster from the highly fragmented MAGs, which are usually generated from highly complex metagenomes using the short-read-based method. So far, investigations of SMs production potential in bacteria and archaea have mainly focused on culturable bacterial isolates and microorganisms from the marine and soil environments [49, 51–53], only limited information is currently available for the AS microbiome. Therefore, in an attempt to bridge this knowledge gap, BGCs were screened and profiled in all 557 MAGs using the genome mining tool antiSMASH [41].

**Fig. 5:**
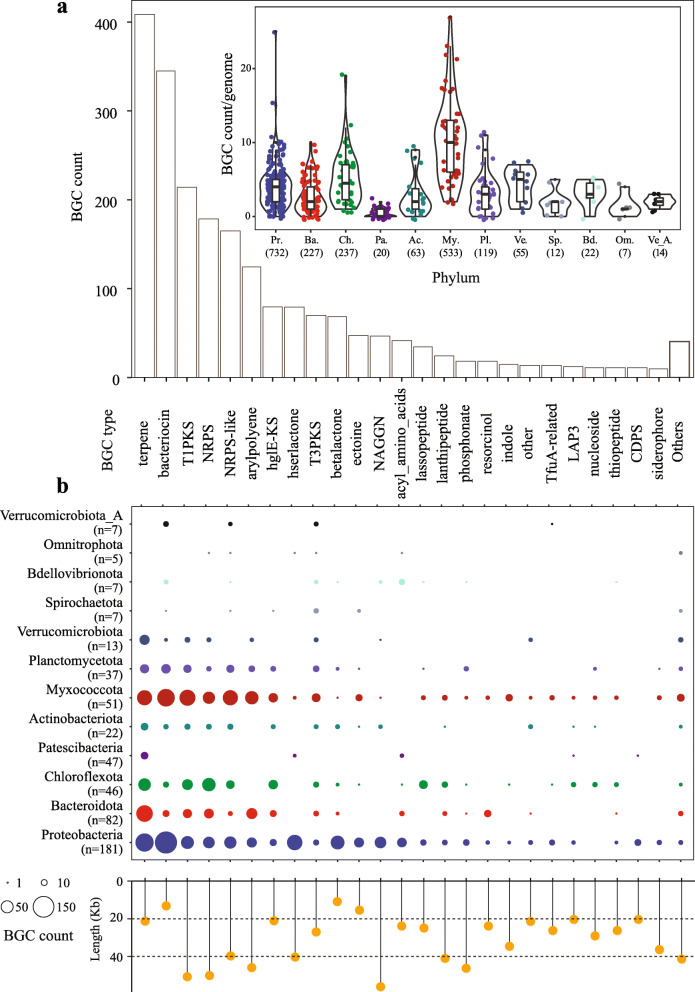
Diversity and distribution of BGCs identified in the recovered activated sludge microbiome. **a** BGC count across different BGC types (outer panel) and distribution of BGC count per genome across various phyla (inner panel). Only the phyla with more than 5 MAGs recovered are shown. In the inner panel, the total number of BGCs identified for each phylum is in brackets. The colors of the dots share the meaning with the colors in **b**. **b** BGC profiles for different phyla of the activated sludge MAGs. The lollipop chart indicates the average length of BGCs identified in our reconstructed MAGs

Overall, 2153 BGCs with 35 BGC types were captured within 481 MAGs (86.4% of the reconstructed MAGs). The majority of these identified BGC types in the AS microbiome were terpene BGC (385), followed by bacteriocin BGC (324), T1PKS BGC (Type I polyketide synthase BGC, 211), and NRPS BGC (Non-ribosomal peptide synthetase cluster BGC, 176) (Fig. 5a and Additional file 1, S5). SMs encoded by these four BGC types are of particular interest since they are utilized by medicine and industry as sources of therapeutic compounds (e.g., antibiotics, immunosuppressants) and environmental restoration (e.g., biosurfactants) [54–57]. Besides, these four BGC types were identified in diverse taxonomic lineages, e.g., *Proteobacteria*, *Myxococcota*, *Bacteroidota*, *Chloroflexota*, *Planctomycetota*, and *Verrucomicrobiota* (Fig. 5b and Additional file 2, Fig. S6), suggesting these microbial taxa have the metabolic potential to produce these metabolites. To avoid bias arising from the genome quantity of different lineages, the occurrence of each BGC type was normalized to the genome count of each lineage. The lineage *Proteobacteria* possessed the most abundant (more than 1/3) identified BGCs (732) with 28 BGC types (Fig. 5), which partly contributed by the largest number of *Proteobacteria* genome (181) reconstructed in this study, because the number of BGC per genome in *Proteobacteria* was not so prominent compared to other phyla (Additional file 2, Fig. S7 and Additional file 1, S5).

The phylum *Myxococcota* including 51 reconstructed MAGs possessed the second largest number of BGCs (533) with 27 BGC types. In particular, *Myxococcota* revealed the highest total BGCs count per genome with an average of 10.5 among the reconstructed AS lineages. We did not attribute the high BGCs count in each MAG to the normally large genome size of *Myxococcota* (the largest genome size was more than 11.0 Mb). Because the average BGCs coding density (gene cluster length/assembled genome size) of *Myxococcota* was also the highest among various phyla, and 4.38 ± 1.89% of reconstructed *Myxococcota* genomes were putatively associated with the biosynthesis of SMs. One genome (HK-STAS-MYXO-17) was observed to include up to 27 BGCs encoding 12 BGC types that were 796 Kb in length. Besides the 4 aforementioned common BGC types, a number of NRPS-like and arylpolyene BGCs was also identified in this lineage (Fig. 5b and Additional file 1, S6). The phylum *Myxococcota* harbored the highest number of BGCs per genome in most identified BGC types compared to other phyla (Additional file 2, Fig. S7), suggesting that *Myxococcota* may serve as a useful microorganism in the future biotechnological development.

Phylogenetic analysis of our reconstructed genomes demonstrated that many identified BGC types within the assembled genomes displayed lineage-specific patterns. Specific BGC types were enriched in certain taxonomic groups. For example, terpene BGCs were the most abundant in *Verrucomicrobiota*. The types of bacteriocin, T1PKS, NRPS-like, and arylpolyene BGCs were well-represented in *Myxococcota*. NRPS BGCs were dominating in *Chloroflexota* (Fig. 5a, Additional file 2, Fig. S7, and Additional file 1, S5).

Since antibiotic producers often encoded antibiotic resistance genes for self-protection [49], we screened and identified 72 antibiotic resistance genes (ARGs) distributed across 63 MAGs (Additional file 1, S6). The majority of the ARG-carrying genomes (95.2%) were found to harbor the capacity to synthesize certain SMs, e.g., enzymes encoded by bacteriocin, T1PKS, NRPS/NRPS-like, and beta-lactone BGCs. (Additional file 2, Fig. S8a). We also found a large proportion of genomes harboring many of the above secondary metabolite potentials but with no identified ARGs (Additional file 2, Fig. S8b); this may suggest the current limitation of the ARGs database and insufficient understanding of resistance mechanisms.

## Discussion

The complexity of environmental samples, including the strain diversity and abundance of the microbial community, influences our ability to reconstruct genomes from metagenomes accurately. Currently, the high demand for computational resources and their large proportion in the total project budget for deeply sequenced, highly complex metagenomes are bottlenecks, limiting our capability to delineate the microbial communities in complex microbiome investigations, e.g., soil, marine, and human gut microbiomes [58, 59]. To provide a workable solution, we proposed the iterative framework to reconstruct reference-quality genomes from highly complex samples.

In addition to the much-improved genome reconstruction performance, the modular characteristic of our workflow enables the full integration of state-of-the-art tools. Furthermore, the haplotype-resolved workflow untangles the complexity of metagenomes by phasing reads into different lineages (candidate bins) and achieving hybrid assembly of microbial populations at the species or strain level. Our study and other works have demonstrated that long reads could empower complete genomes reconstruction from metagenomes [2, 18, 24]. The proposed iterative strategy [24] and haplotype-resolved method [60] could provide additional insights into launching the new era of “complete metagenomics” [61], enabling more circular, closed genomes to be deposited into reference databases [18].

The collected 125 HQ/complete MAGs harbored full-length rRNA genes, accounting for nearly 30% of the Shatin AS microbial community. Notably, full-length 16S rRNA genes were identified within 410 MAGs, representing 47.5% microbial community (Additional file 1, S3). Linking 16S rRNA genes to the reconstructed MAGs enables precise prediction of potential functional profiles when relying on the cost-effective amplicon sequencing to monitor microbial communities in the local WWTP, especially considering the poor representation of high-quality AS genomes in public reference databases [62] and high diversity of the AS microbiome [13]. The reconstructed high-quality genomes will also help to improve functional prediction accuracy within similar lineages.

We reconstructed multiple highly contiguous and accurate MAGs from the AS system, allowing us to profile the microbial community and potential metabolic traits in high resolution. Genome-centric analysis reveals the prevalence of truncated pathways within the reconstructed AS microbiome. For instance, microbes harboring the incomplete denitrification pathway are more than those encoding the complete pathway (Fig. 3c). Cross-feeding benefits among microbes have been demonstrated in previous studies [63–65]; the synergistic network may promote the growth of relevant cohorts in the microbial community. Nonetheless, how the truncated and complete pathways affect the resistance and resilience of the microbial community under disturbance remains unclear. Additionally, although many genes encoded by MAGs are involved in the undesired process or intermediate products’ generation in the WWTP, e.g., the nitrite ammonification process and the release of N_2_O (Fig 4b) [66, 67], the expression and impact of these transformations require further investigation.

To the best of our knowledge, the current study demonstrated the occurrence and distribution of BGCs harbored in the AS microbiome for the first time. The observed highly abundant and diverse BGCs identified in the reconstructed high-contiguity MAGs suggested that the AS microbiome might be a pristine treasure for the discovery of novel and valuable microbial bio-products. Besides, the lineage-specific analysis of BGCs provides potential phylogenetic targets for the generation of secondary metabolites of interest. However, the potential versatility of the AS microbiome was only demonstrated based on reconstructed MAGs and many BGCs are presumably inactive [68, 69], confident links between diverse identified BGCs to the production of secondary metabolites require further metatranscriptomics and chemical screening (e.g., liquid chromatography-mass spectrometry) lines-of-evidence. Furthermore, induction mechanisms of these silent BGCs within the AS microbial consortia should also be discovered for developing new biotechnological and medical applications in the future. Considering that AS is a highly populated environment, microbial interaction may be more effective than in other dilute settings. Thus, investigation of BGCs in diverse environments may provide new insights into intracellular and intercellular roles of these secondary metabolites in nature [53].

Our iterative haplotype-aware approach recovered many (near-) complete MAGs from the highly complex AS metagenome; some previously known issues might remain, such as chimeric bins and inaccessible genome gaps. Thus, biological knowledge of specific populations and individual detailed manual curations might be required [70], although largely reduced. Besides, a significant proportion of microbial communities (~ 40%) were still not recovered; therefore, enrichment or isolation efforts should be considered to approach the comprehensive picture of the AS microbiome.

## Conclusions

Overall, the iterative haplotype-resolved HCBHA approach developed addressed the challenge of HQ and high-contiguity (even complete) genome reconstruction from deeply sequenced, highly complex metagenomes. The high-contiguity MAGs greatly advance our capacity to capture the signals of some determinants, e.g., BGCs, allowing rapid and detailed screening of functional potential for the microbial community from complex environments. The wealth of identified SM biosynthetic potential reveals that the AS microbiome might be an untapped reservoir for discovering novel natural products, e.g., new antibiotics. Further experiments will be required to validate and decipher the functions of the SMs harbored in the AS microbiome.

## Supplementary Information

**Additional file 1: S1.** Summary of the sequencing dataset in the present study. **S2.** Performance evaluation of different hybrid assembly workflows using the Mock and Mock-STAS dataset. **S3.** Genome features for 557 MAGs recovered from the highly-sequenced ST-AS samples using the iterative Hierarchical Clustering Based Hybrid Assembly (HCBHA) approach developed in the present study. Taxonomic assignments were identified using GTDB-Tk. **S4**. Metabolic functional trait profile to the reconstructed 557 MAG dataset. **S5.** Biosynthetic gene clusters (BGCs) identified in the reconstructed 557 MAG dataset using antiSMASH. **S6.** Biosynthetic gene clusters (BGCs) profile in the antibiotic resistance genes (ARG)-carrying MAGs.

**Additional file 2: **Methods. **Fig. S1**. Genome quality evaluation of hybrid assembly workflows. **Fig. S2.** Assemblies comparison among three activated sludge studies. **Fig. S3.** Phylogeny of the recovered 552 bacterial genomes in the Shatin activated sludge. **Fig. S4.** Taxonomic distribution of reconstructed high-quality/complete MAGs. **Fig. S5.** Metabolisms associate with sulfur cycle in the wastewater treatment plant. **Fig. S6.** Association networks between biosynthetic gene clusters and the corresponding lineages. **Fig. S7.** Distribution of the biosynthetic gene clusters identified across the dominant taxonomic groups. **Fig. S8.** Association networks between genomes with (a) / without (b) identified antibiotic resistance genes and the diverse biosynthetic gene clusters.

## Data Availability

The raw DNA sequences and the reconstructed genomes in the present study have been deposited in the NCBI database with BioProject accession number PRJNA648801, as well as the time serious AS data in our previous study under the NCBI project PRJNA432264.

## Abbreviations

HCBHA: Hierarchical clustering-based hybrid assembly
AS: Activated sludge
MAGs: Metagenome-assembled genomes
WWTP: Wastewater treatment plant
BGCs: Biosynthesis gene clusters
ORF: Open reading frame
SARG: Structured antibiotic resistance gene
AGF: Aligned genome fraction
T1PKS: Type I Polyketide synthase
NRPS: Non-ribosomal peptide synthetase cluster
*hox*: Hydrogen oxidation
