# Supplementary material for: Charting the complexity of the activated sludge microbiome through a hybrid sequencing strategy
Source: Microbiome. 2021 Oct 15;9:205. doi: 10.1186/s40168-021-01155-1 (PMC8518188; doi:10.1186/s40168-021-01155-1)
Supplement: Supplementary file 3 — Additional file 2: Methods. Fig. S1. Genome quality evaluation of hybrid assembly workflows. Fig. S2. Assemblies comparison among three activated sludge studies. Fig. S3. Phylogeny of the recovered 552 bacterial genomes in the Shatin activated sludge. Fig. S4. Taxonomic distribution of reconstructed high-quality/complete MAGs. Fig. S5. Metabolisms associate with sulfur cycle in the wastewater treatment plant. Fig. S6. Association networks between biosynthetic gene clusters and the corresponding lineages. Fig. S7. Distribution of the biosynthetic gene clusters identified across the dominant taxonomic groups. Fig. S8. Association networks between genomes with (a) / without (b) identified antibiotic resistance genes and the diverse biosynthetic gene clusters. [file 40168_2021_1155_MOESM3_ESM.docx]

**Supplementary Information for**

**Charting the complexity of the activated sludge microbiome through a hybrid sequencing strategy**

Lei Liu^1,2,3^, Yulin Wang^1^, Yu Yang^1^, Depeng Wang^4^, Suk Hang Cheng^5^, Chunmiao Zheng^2,3^, Tong Zhang^1,3^*

^1^Environmental Microbiome Engineering and Biotechnology Laboratory, The University of Hong Kong, Hong Kong SAR, China.

^2^State Environmental Protection Key Laboratory of Integrated Surface Water-Groundwater Pollution Control, School of Environmental Science and Engineering, Southern University of Science and Technology, Shenzhen, China.

^3^School of Environmental Science and Engineering, Southern University of Science and Technology, Shenzhen, China.

^4^Nextomics Biosciences Institute, Wuhan, China.

^5^Department of Chemical Pathology, The Chinese University of Hong Kong, Hong Kong SAR, China.

Corresponding author: Tong Zhang; Phone: +85228578551; E-mail: zhangt@hku.hk

**This file (Additional file 2) includes:**

Methods

Figures S1 to S8

Legends for Datasets S1 to S6

**Other supplementary materials (Additional file 1) for this manuscript include the following:**

Sheet S1 to S6

## Methods

### Hybrid assembly workflow for high-complexity ecosystems

The Hierarchical Clustering Based Hybrid Assembly (HCBHA) workflow integrated multiple state-of-the-art bioinformatic tools and enables the high-quality/complete genome reconstruction from highly complex environmental samples. In Step 1, both Illumina short reads and Oxford Nanopore Technology (ONT) long reads are required.

Step 2 **Long-read *de novo* assembly**. Error-prone long reads were assembled using a long-read assembler, Flye v2.4.2 [1], to rapidly generate the high-contiguity assemblies’ drafts with the parameters “--nano-raw -i 5 -g 4m --meta”. At this step, the nucleotide accuracy of assemblies was lower than that of the short-read assembled contigs.

Step 3 **Initial binning**. Short-read sequences from two different commercial kits were selected to provide the necessary microbial genome signatures [2] and cluster the Flye assemblies into ‘raw bin clusters’ using MetaWRAP (v1.1) with ‘binning (--metabat2 [3] and --maxbin2 [4])’ and ‘bin_refinement (-c 50 -x 10)’ modules [5]. Resulting from the relatively low nucleotide accuracy of the assemblies, the majority of the bins do not pass the quality filtering screened by CheckM (v1.0.8) [6], the ‘refined bins’ generated from the MetaWRAP are selected for the ‘raw bin clusters’.

Step 4 **Initial bins polish**. The long-read sequences and short-read sequences were mapped to all collected ‘raw bin clusters’ using minimap2 (2.17-r941) [7] with parameters ‘-x map-ont’ and ‘-x sr’, respectively. All mapped long reads assigned ‘raw bin cluster’ were further filtered at cutoffs of at least 70% similarity and 70% coverage, while the short reads were filtered at cutoffs of at least of 80% similarity and 80% coverage. The filtered long and short reads were then collected as ‘long-read cluster’ and ‘short-read cluster’ using seqtk (v1.3) and GNU Parallel (20161222) [8], which could be considered as sequencing reads of bacterial or archaeal isolates to some extent. Each short-read and long-read cluster pair then were subjected to a hybrid assembly process using Unicycler (v0.4.8-beta) [9] with parameter ‘-1 -2 -l --no_correct --min_fasta_length 1000’. By doing so, the assemblies in each ‘raw bin cluster’ were polished and the nucleotide accuracy increased as well.

Step 5 **Re**-**binning**. This step aims to use the corrected contigs signals and avoid the chimera bins induced by long-read assembly of the high complex metagenome. The entire polished ‘raw bin clusters’ were combined and the second binning process was performed using MetaWRAP [5] with the same parameters in Step 3, while ‘candidate bins’ with completeness of more than 50% and contamination of less than 10% were passed on to the next step.

Step 6 **Re-assembly**. Similar to Step 4, long-read and short-read data were mapped on to the ‘candidate bins’. Then the long-read and short-read cluster pairs were identified with the same parameters used in Step 4. Unicycler [9] was used again to reassembly each ‘candidate bins’ to bridge the gaps and further improve the contiguity of the bins. Unicycler was also used to confirm whether the genome was circular. Reassembly process which used to improve the genome contiguity has also been demonstrated in the program MetaWRAP [5].

Step 7 **Final binning**. After the Re-assembly step, the features of some contigs might change. To remove genome contaminations and fully take advantage of more accurate contiguous contigs, all the reassembled ‘candidate bins’ were combined and the final binning process was performed, similar to Step 5. This step aimed to generate high-accuracy and high-contiguity MAGs.

Notably, in the iterative genome reconstruction process, the MQ MAGs retrieved in the former iteration had the chance to be re-assembled into HQ ones due to the decreased assembly inference and increased sequencing coverage in the latter metagenomic assembly dataset.

### Lineage-resolved clustering based HCBHA approach to further improve genome reconstruction from the activated sludge sample

Besides removing reads assigned to the qualified MAGs from the total sequences, we also performed another HCBHA cycle which applying lineage-based MAGs clustering, i.e., if the completeness of MAGs in our reconstructed genome dataset is better than the representative genome of a certain lineage in a GTDB (RS89) [10], then reads assigned to these MAGs would also be removed from the total sequences. However, this process showed little improvement on the genome reconstruction from the AS sample, mostly due to the low relative abundance of the selected MAGs.

### Relative abundance estimation of reconstructed MAGs

The relative abundances of the reconstructed 557 MAGs based on read mapping were calculated by coverm (v0.4.0, https://github.com/wwood/CoverM) with arguments ‘coverm genome -m relative_abundance --min-read-percent-identity 95 --min-read-aligned-percent 75 --min-covered-fraction 0.1’ [11] and with the mapper minimap2 [7]. Additionally, both Bowtie2 [12] based on the mapped reads with the parameter ‘--very-sensitive’ and singleM (v0.13.2, https://github.com/wwood/singlem) based on the 14 single copy marker genes with default parameters were used to determine the community recovery rate. SingleM was also tried to discover the unbinned community in the AS system.

### Phylogenetic tree construction

Because we retrieved 5 archaeal genomes from the same class (*Nanoarchaeia*), the phylogenetic analysis only contained 552 bacterial genomes. The tool GTDB-Tk (v1.1.0, gtdbtk *de*_*novo*_wf) [13] was performed using 120 bacterial maker genes and GTDB RS89 [10] to build a genome phylogeny with third-party dependencies pplacer (v1.1), Prodigal (V2.6.3) [14], FastTree 2 (v2.1.10) [15] and mash (v2.1) [16]. The tree file was imported into the online website iTOL (https://itol.embl.de/) [17] for visualization.

### Secondary metabolite biosynthesis gene clusters (BGCs) identification

Secondary metabolite biosynthesis gene clusters (BGCs) were identified using antiSMASH (v5.0) [35] with full featured analysis (--cb-general --cb-knownclusters --cb-subclusters --asf --pfam2go --smcog-trees). “Others” indicated a collection of some rare BGC types identified in the present AS ecosystem., including those clusters when expressed synthesizing butyrolactone, cyanobactin, linaridin, oligosaccharide, Polyunsaturated fatty acid cluster (PUFA), transAT-PKS, proteusin, ladderane, microviridin and other types of polyketide synthase (PKS) cluster (PKS-like).

**
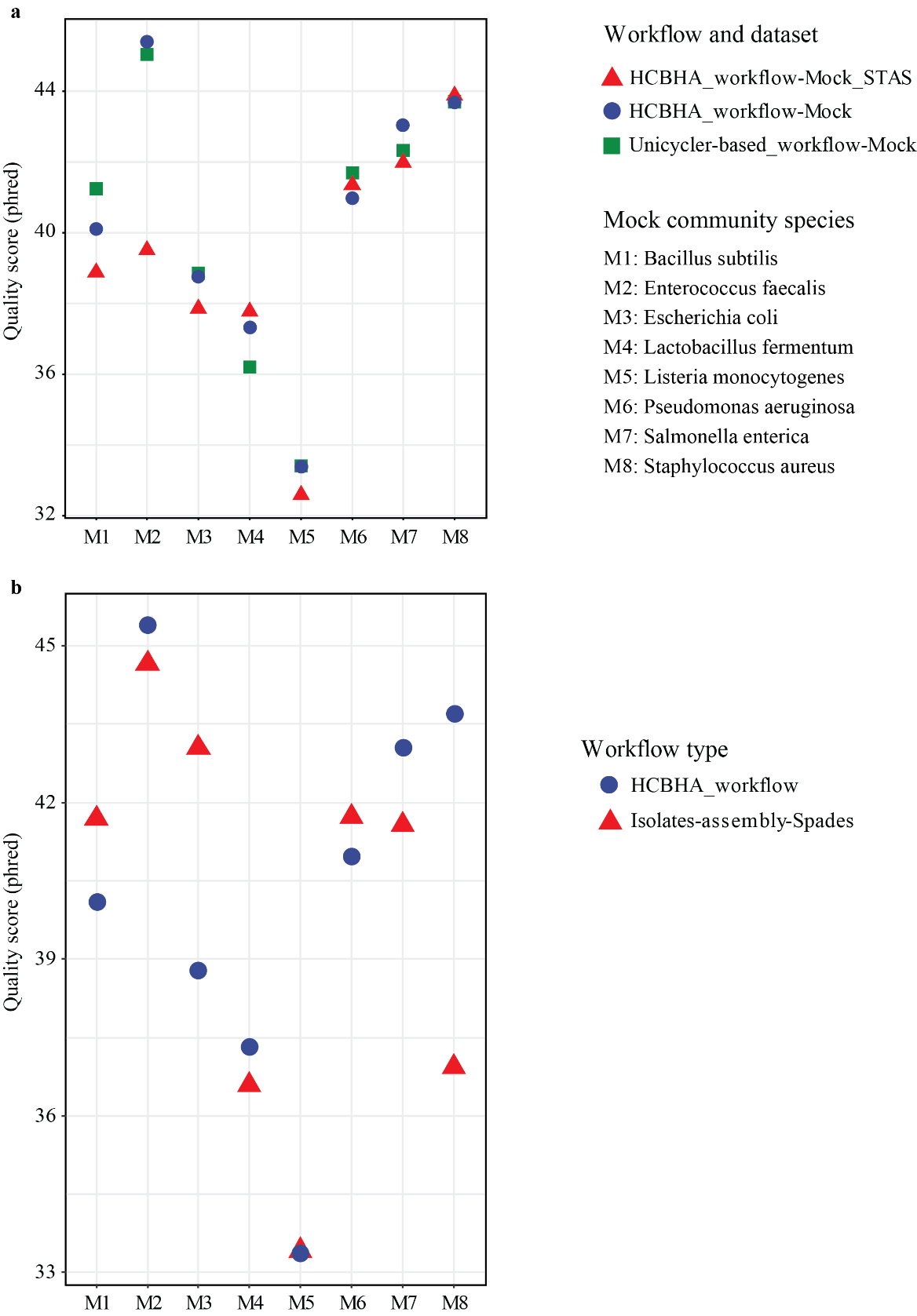
**

**Fig. S1 Genome quality evaluation of hybrid assembly workflows.** **a,** Genome quality comparison using two hybrid assembly workflows under two constructed datasets scenarios. HCBHA indicated the method proposed in this study. Unicycler-based workflow indicated the method that directly performs hybrid assembly using Unicycler. **b,** Assembled genome quality comparison between hybrid assembly workflow for high-complexity metagenome and individual genome assembly using SPAdes.

**
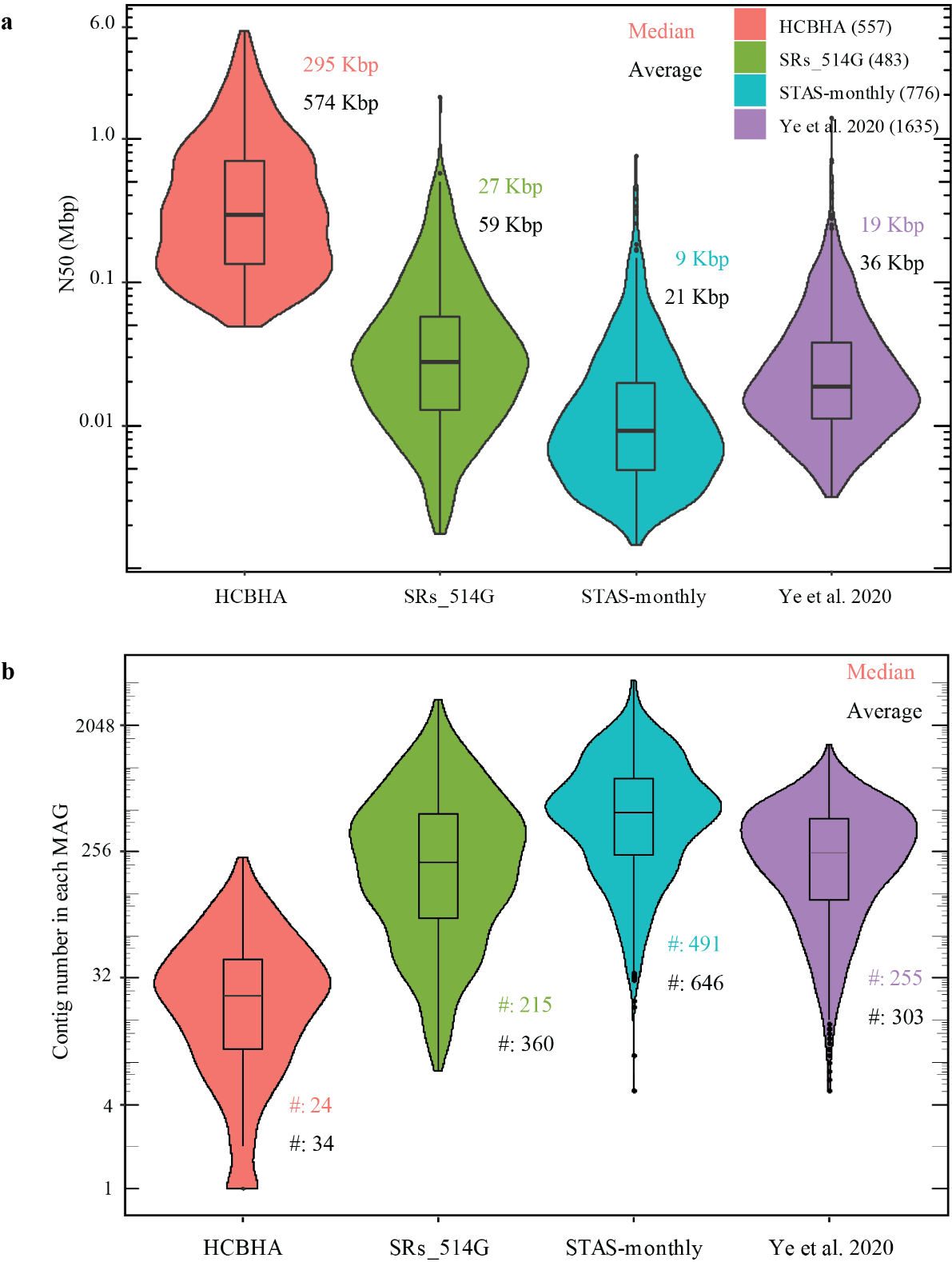
**

**Fig. S2 Assemblies comparison among three activated sludge studies.** ‘HCBHA’ stands for the approach used in this study. SRs_514G shows the results from short-read-based method using concatenated short-read dataset with 514 Gbp involved. ‘STAS-monthly’ indicates the genomes from a 9-year time serious short-read dataset (not published). ‘Ye et al. 2020’ presents the genome statistics from the largest activated sludge metagenomic studies so far. All genomes were dereplicated again using dRep with the parameters selected in this study. Numbers in the parentheses indicates the dereplicated MAGs number. **a,** N50 comparison of assembled MAGs. **b,** Contig number comparison of assembled MAGs.


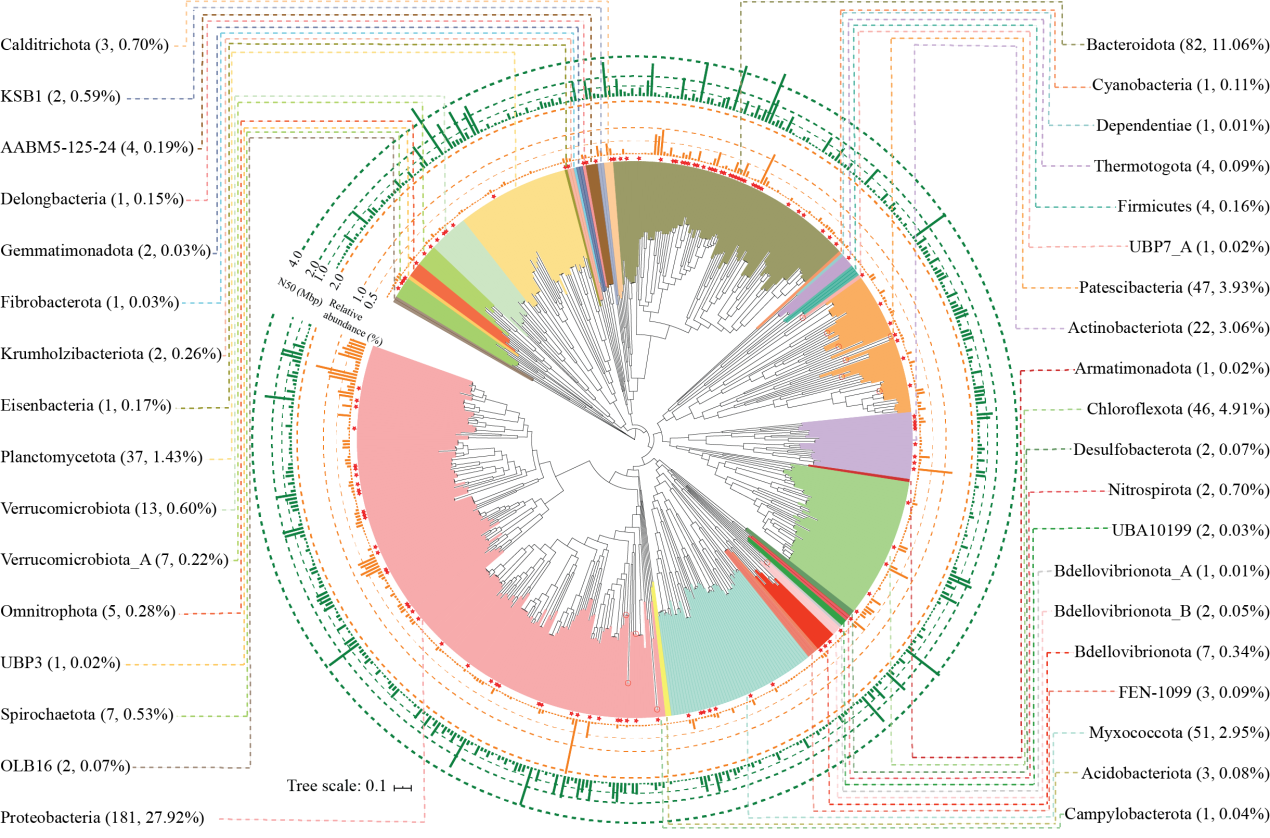


**Fig. S3 Phylogeny of the recovered 552 bacterial genomes in the Shatin activated sludge.** A phylogenetic tree is inferred using GTDB-Tk. Red stars show the high-quality/complete genomes reconstructed. Numbers in parentheses presented the total number of the recovered MAGs and their cumulative relative abundance in the phylum. The relative abundance and N50 of each reconstructed MAG are indicated by the orange and dark-cyan barcharts, respectively.

**
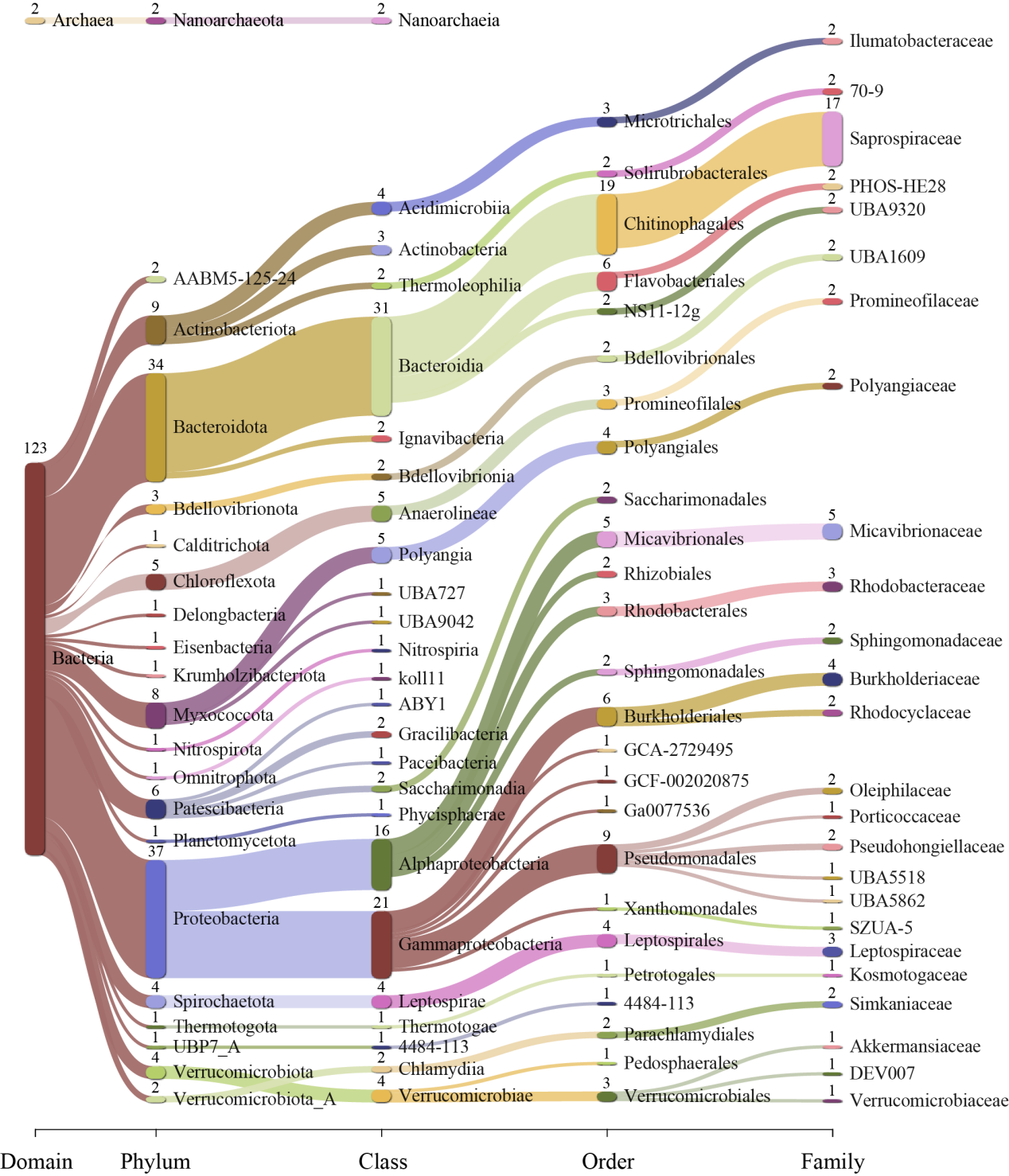
**

**Fig. S4 Taxonomic distribution of reconstructed high-quality/complete MAGs.** Only top 25 taxa in each lineage are shown and plot using online tool Pavian (https://fbreitwieser.shinyapps.io/pavian/). Numbers shows the recovered high-quality MAGs count.

**
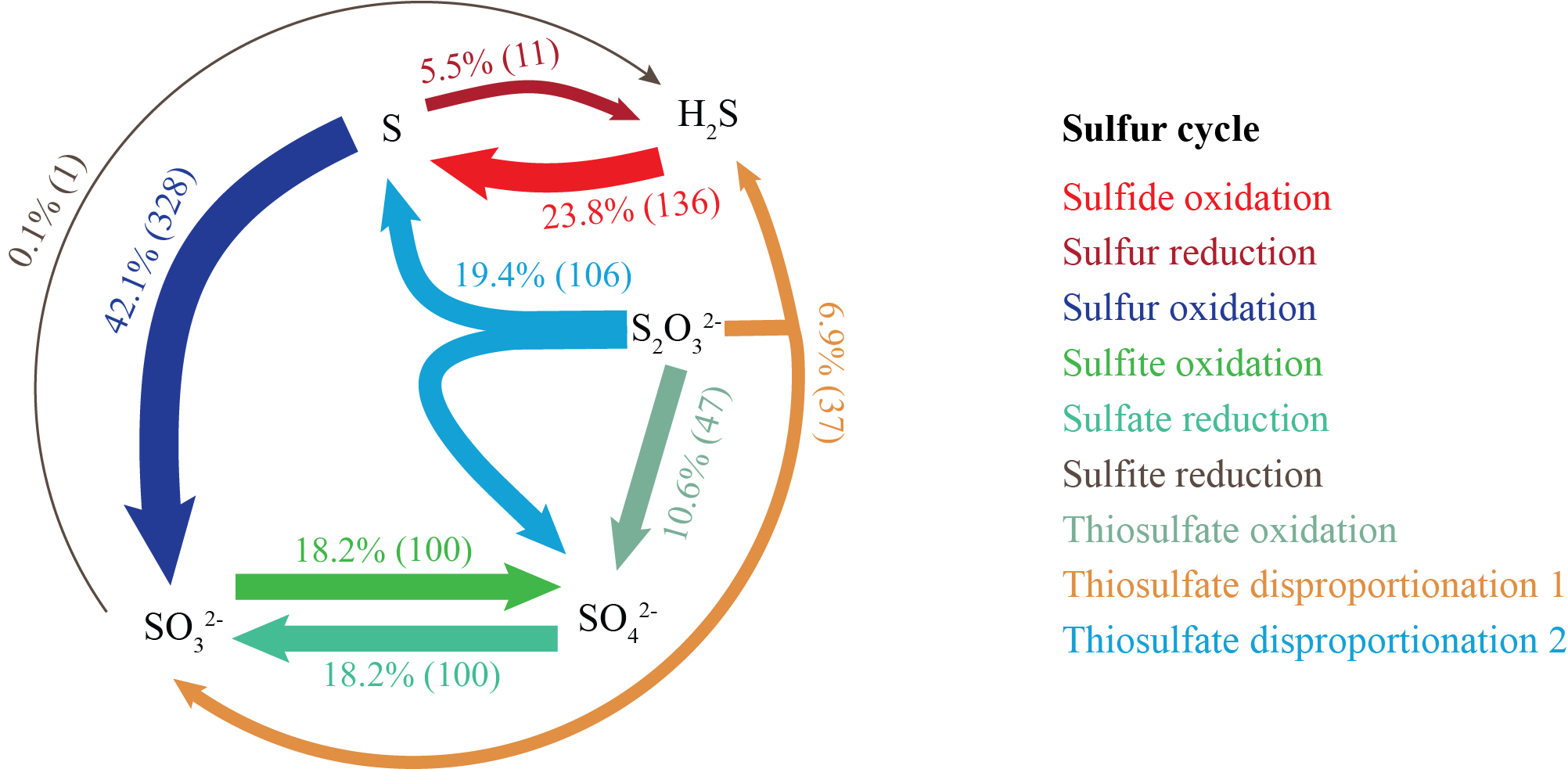
**

**Fig. S5 Metabolisms associate with sulfur cycle in the wastewater treatment plant.** Percentages and numbers stand for the relative abundance of MAGs and the MAGs count involved in the process, respectively.

**
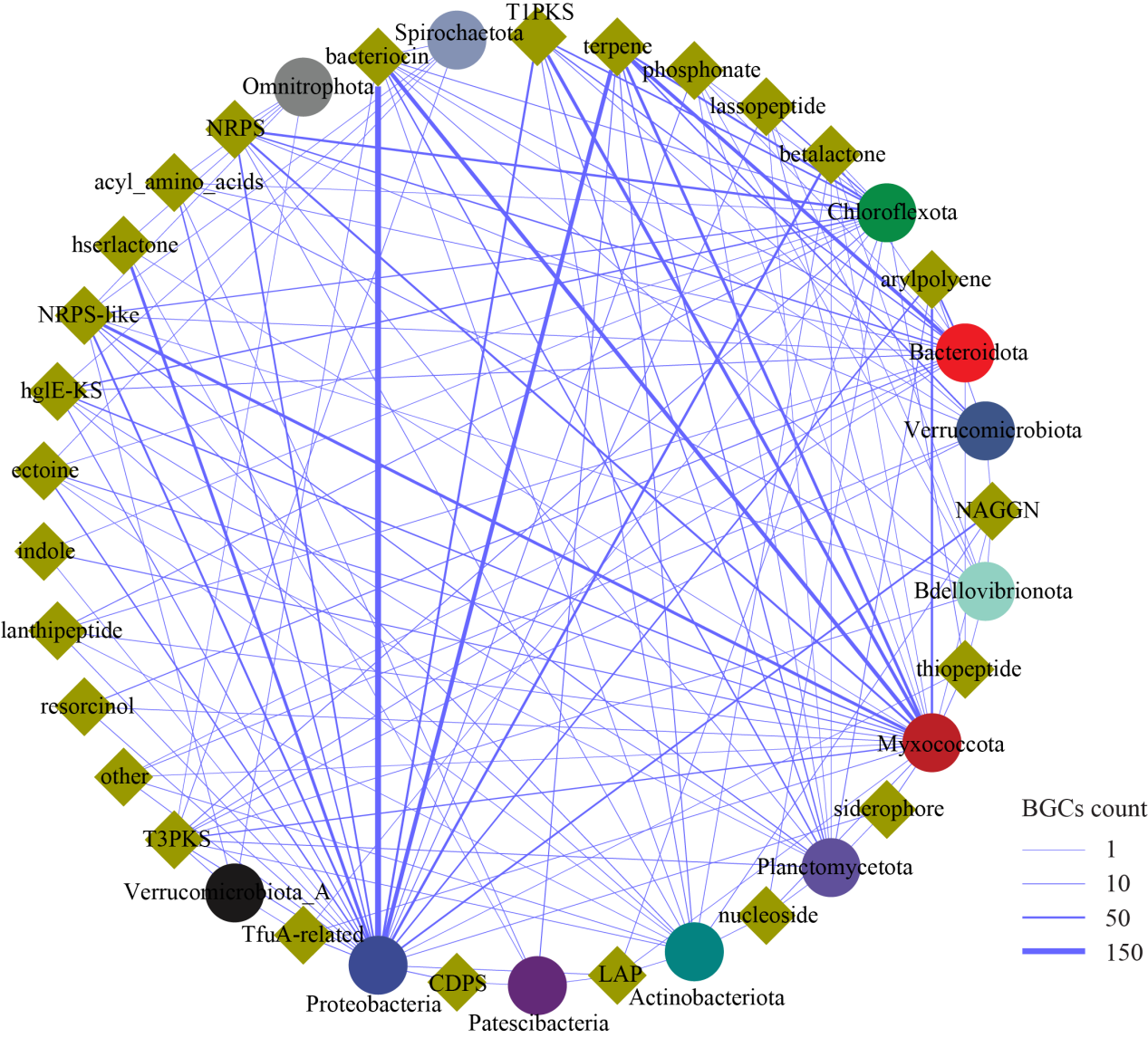
**

**Fig. S6 Association networks between biosynthetic gene clusters and the corresponding lineages.** The thickness of the line indicat the biosynthetic gene clusters number identified by the lineage.

**
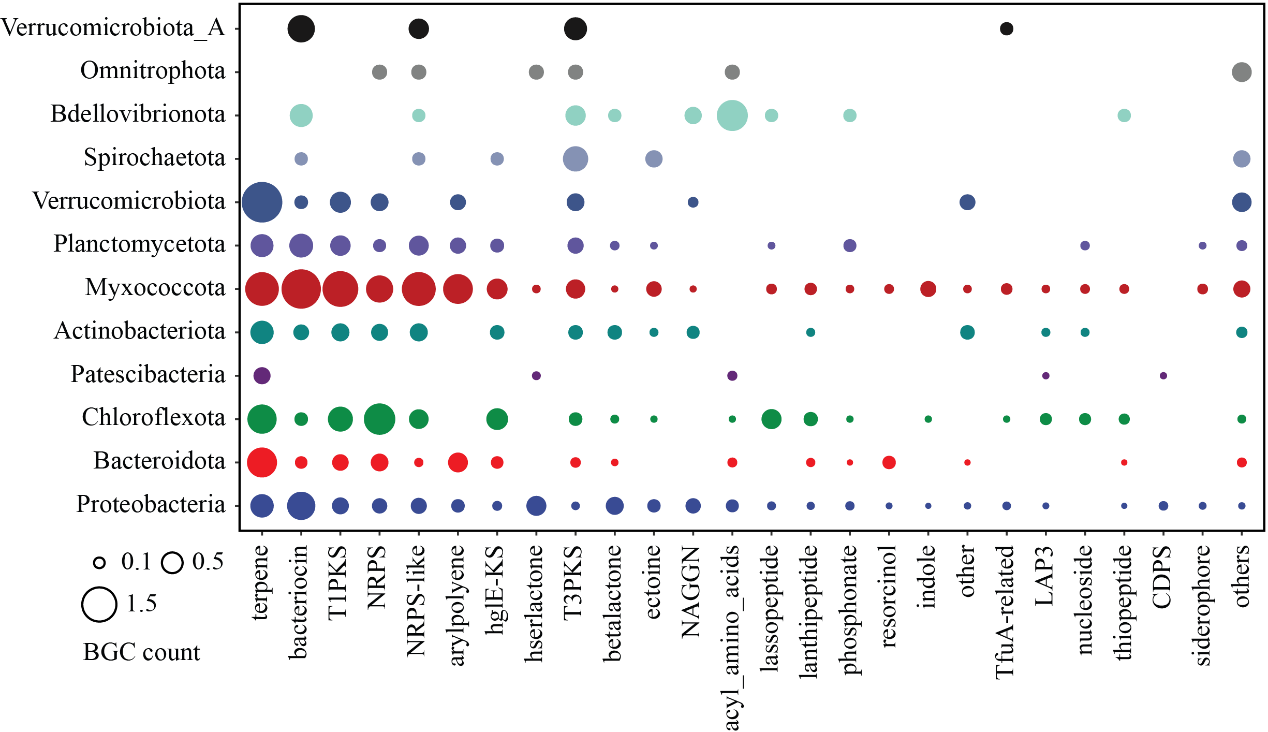
**

**Fig. S7 Distribution of the biosynthetic gene clusters identified across the dominant taxonomic groups.** The biosynthetic gene cluster types are derived from classifications of antiSMASH and cluster counts were normalized to per genome.

**
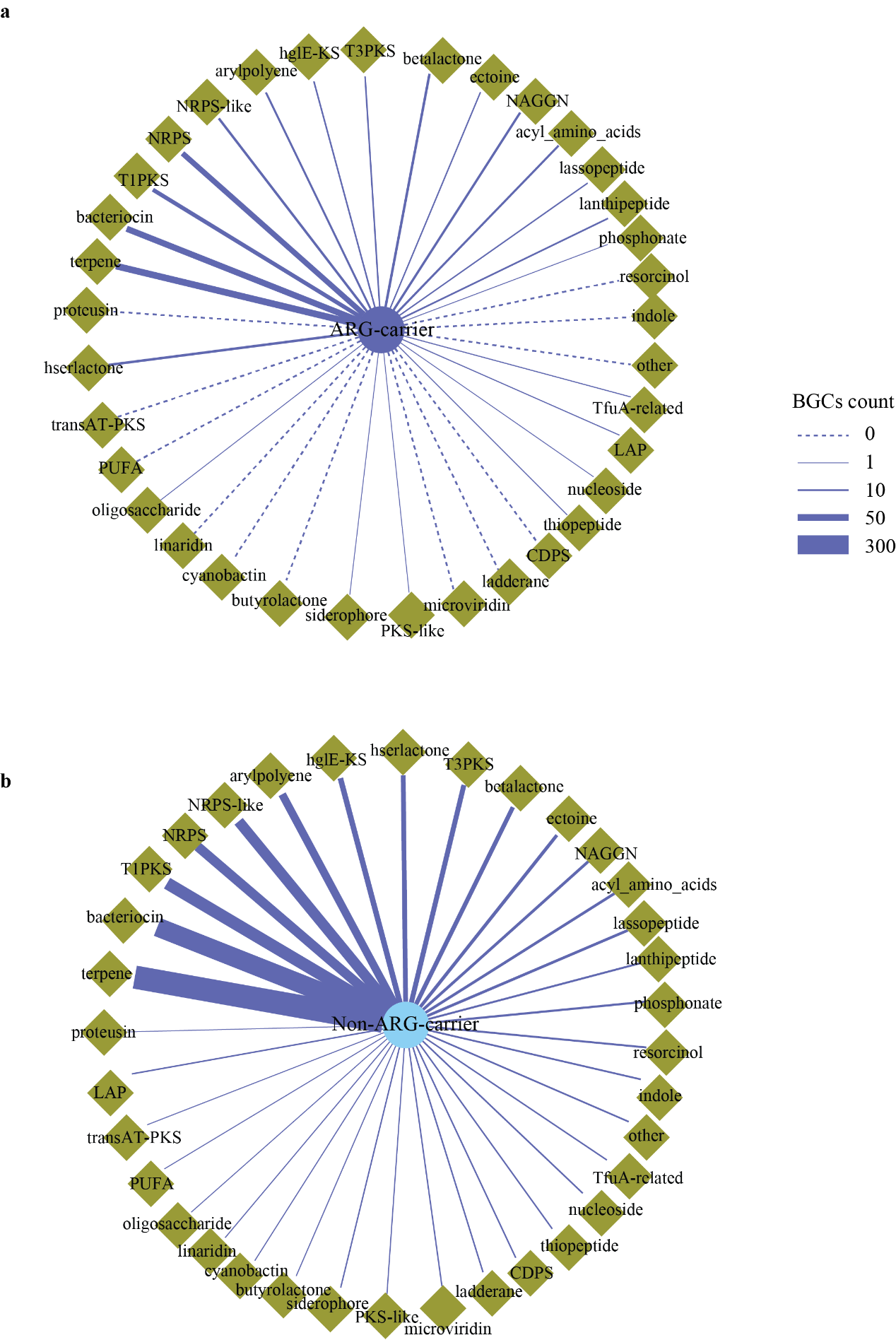
**

**Fig. S8 Association networks between genomes with (a) / without (b) identified antibiotic resistance genes and the diverse biosynthetic gene clusters.**

**Additional file 1, S1**: Summary of the sequencing dataset in the present study.

**Additional file 1, S2**: Performance evaluation of different hybrid assembly workflow using the Mock and Mock-STAS dataset.

**Additional file 1, S3**: Genome features for 557 MAGs recovered from the highly-sequenced ST-AS samples using the iterative Hierarchical Clustering Based Hybrid Assembly (HCBHA) approach developed in the present study. Taxonomic assignments were identified using GTDB-Tk.

**Additional file 1, S4**: Metabolic functional trait profile to the reconstructed 557 MAG dataset.

**Additional file 1, S5**: Biosynthetic gene clusters (BGCs) identified in the reconstructed 557 MAG dataset using antiSMASH.

**Additional file 1, S6**: Biosynthetic gene clusters (BGCs) profile in the antibiotic resistance genes (ARG)-carrying MAGs.

## References

1. Kolmogorov, M., J. Yuan, Y. Lin, and P.A. Pevzner, *Assembly of long, error-prone reads using repeat graphs.* Nat Biotechnol, 2019. **37**(5): p. 540-546.

2. Albertsen, M., P. Hugenholtz, A. Skarshewski, K.L. Nielsen, G.W. Tyson, and P.H. Nielsen, *Genome sequences of rare, uncultured bacteria obtained by differential coverage binning of multiple metagenomes.* Nat Biotechnol, 2013. **31**(6): p. 533-8.

3. Kang, D.D., J. Froula, R. Egan, and Z. Wang, *MetaBAT, an efficient tool for accurately reconstructing single genomes from complex microbial communities.* PeerJ, 2015. **3**: p. e1165.

4. Wu, Y.-W., B.A. Simmons, and S.W. Singer, *MaxBin 2.0: an automated binning algorithm to recover genomes from multiple metagenomic datasets.* Bioinformatics, 2015. **32**(4): p. 605-607.

5. Uritskiy, G.V., J. DiRuggiero, and J. Taylor, *MetaWRAP-a flexible pipeline for genome-resolved metagenomic data analysis.* Microbiome, 2018. **6**(1): p. 158.

6. Parks, D.H., M. Imelfort, C.T. Skennerton, P. Hugenholtz, and G.W. Tyson, *CheckM: assessing the quality of microbial genomes recovered from isolates, single cells, and metagenomes.* Genome Res, 2015. **25**(7): p. 1043-55.

7. Li, H., *Minimap2: pairwise alignment for nucleotide sequences.* Bioinformatics, 2018. **34**(18): p. 3094-3100.

8. Tange, O., *Gnu parallel-the command-line power tool.* The USENIX Magazine, 2011. **36**(1): p. 42-47.

9. Wick, R.R., L.M. Judd, C.L. Gorrie, and K.E. Holt, *Unicycler: Resolving bacterial genome assemblies from short and long sequencing reads.* PLoS Comput Biol, 2017. **13**(6): p. e1005595.

10. Parks, D.H., M. Chuvochina, P.-A. Chaumeil, C. Rinke, A.J. Mussig, and P. Hugenholtz, *A complete domain-to-species taxonomy for Bacteria and Archaea.* Nature Biotechnology, 2020: p. 1-8.

11. Singleton, C.M., F. Petriglieri, J.M. Kristensen, R.H. Kirkegaard, T.Y. Michaelsen, M.H. Andersen, Z. Kondrotaite, S.M. Karst, M.S. Dueholm, and P.H. Nielsen, *Connecting structure to function with the recovery of over 1000 high-quality activated sludge metagenome-assembled genomes encoding full-length rRNA genes using long-read sequencing.* bioRxiv, 2020.

12. Langmead, B. and S.L. Salzberg, *Fast gapped-read alignment with Bowtie 2.* Nat Methods, 2012. **9**(4): p. 357-9.

13. Chaumeil, P.A., A.J. Mussig, P. Hugenholtz, and D.H. Parks, *GTDB-Tk: a toolkit to classify genomes with the Genome Taxonomy Database.* Bioinformatics, 2019.

14. Hyatt, D., G.-L. Chen, P.F. LoCascio, M.L. Land, F.W. Larimer, and L.J. Hauser, *Prodigal: prokaryotic gene recognition and translation initiation site identification.* BMC bioinformatics, 2010. **11**(1): p. 119.

15. Price, M.N., P.S. Dehal, and A.P. Arkin, *FastTree 2–approximately maximum-likelihood trees for large alignments.* PloS one, 2010. **5**(3): p. e9490.

16. Ondov, B.D., T.J. Treangen, P. Melsted, A.B. Mallonee, N.H. Bergman, S. Koren, and A.M. Phillippy, *Mash: fast genome and metagenome distance estimation using MinHash.* Genome Biol, 2016. **17**(1): p. 132.

17. Letunic, I. and P. Bork, *Interactive Tree Of Life (iTOL) v4: recent updates and new developments.* Nucleic acids research, 2019. **47**(W1): p. W256-W259.
